# Intramucosal leiomyosarcoma of the stomach following hereditary retinoblastoma in childhood – a case report and review of the literature

**DOI:** 10.1186/1477-7819-6-131

**Published:** 2008-12-14

**Authors:** Ursula Pauser, Horst Grimm

**Affiliations:** 1 Department of Pathology, University of Lübeck, Lübeck, Germany; 2 Department of Endoscopic Surgery, University of Kiel, Kiel, Germany

## Abstract

**Background:**

Leiomyosarcomas of the stomach are very rare. At the time of primary diagnosis the tumors are most often in advanced stage and the patients complain of abdominal pain due to large tumor size. Endosonographically, the tumors impress as submucous mass with suspicion to malignancy. Sarcomas following hereditary retinoblastoma in childhood are in generally located in the soft tissue. Structural alterations of the *retinoblastoma *gene (*RB1*) seem to be involved in the pathogenesis.

**Case presentation:**

A 37-year-old german male suffered from reflux disorder. In endoscopic examination a small polypous tumor was detected in the stomach. The resection specimen revealed an intramucosal leiomyosarcoma. At the age of one year, the patient had a retinoblastoma.

**Conclusion:**

This is the unique report of an intramucosal gastric leiomyosarcoma and the first account of a gastric leiomyosarcoma after retinoblastoma in childhood. A careful clinical follow-up is advised because of increased risk of developing further metachronous malignancies.

## Background

Leiomyosarcomas are of smooth muscle origin and develop in the soft tissue of the vessel wall and in the smooth muscle layer of visceral organs. In the gastrointestinal tract, they normally arise in the submucosa and bulge out the mucosa and serosa. They present most often in advanced tumor stage. In this case we report of an intramucosal gastric leiomyosarcoma. Medical history referred treatment of a retinoblastoma in childhood. Sarcomas following hereditary retinoblastoma are in generally located in the soft tissue. Structural alterations of *RB1 *seem to be involved in the pathogenesis of the secondary malignancy after treatment of retinoblastoma.

## Case presentation

A 37-year-old man with reflux symptomatic was sent to endoscopic examination. During the examination strabismus was striking. The patient reported about eye operation with 1 year of age, due to a retinoblastoma. Both eyes were affected. The right eye was enucleated. The left eye was treated with laser. There was no tumor relapse. A germline mutation in *RB1 *was detected in 1988. In family history there is no further case of retinoblastoma. Due to tumor prevention the patient underwent endoscopic examination. In gastroscopy, a 1 cm in diameter polypous lesion was found in the antrum of the stomach. It was suspicious to be a hyperplastic polyp or an adenoma of gastric mucosa. The polypous lesion was resected endoscopically and was sent to histopathological investigation. Representative 4 μm sections of formalin-fixed, paraffin-embedded tissue from the tumor specimens were stained with hematoxylin and eosin (H&E) and periodic acid-Schiff. Immunohistochemical staining was performed using the standard avidin-biotin method with antibodies against smooth muscle actin (SMA, 1:20, Dako Cytomation, Glostrup, Denmark), S100 (1:500, Dako Cytomation), CD34 (1:500, Immunotech, Marseille, France) and KIT (1:50, Dako Cytomation). The proliferative activity was assessed by staining the tissue with the antibody MiB-1 (1:100, Dako Cytomation).

**Figure 1:**
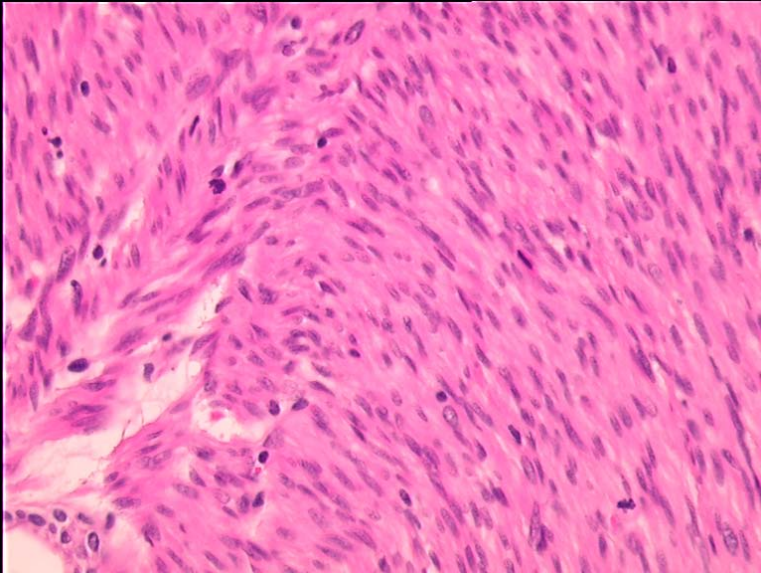
**Tumor infiltrate with spindle cells arranged in parallel bundles**. The nuclei were elongated with plump ends and focally mild atypia. Several mitoses are shown (HE, original magnification × 400).

**Figure 2:**
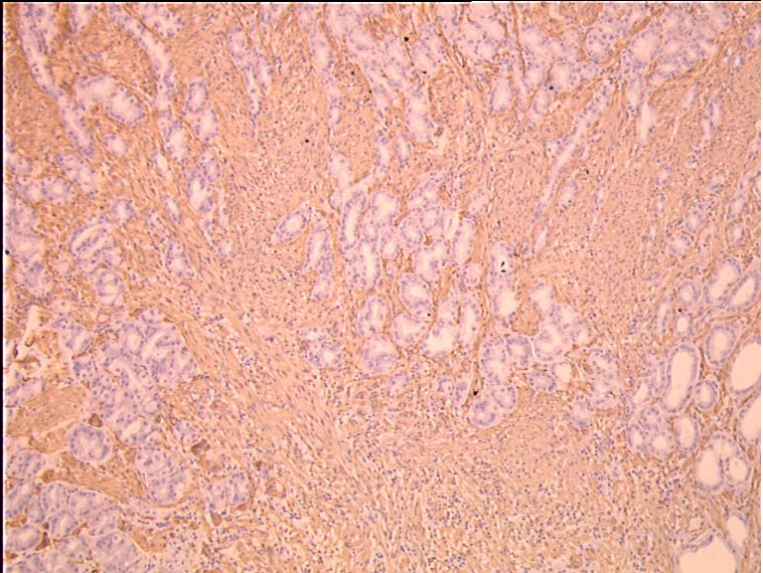
**Immunohistochemical staining highlight the diffuse spindle cell infiltrate in the gastric mucosa**. The tumor infiltrates the stroma between regular differentiated gastric glands. The tumor cells stain strongly positive for SMA. The preexisting gastric glands are negative (SMA, original magnification × 100).

**Figure 3:**
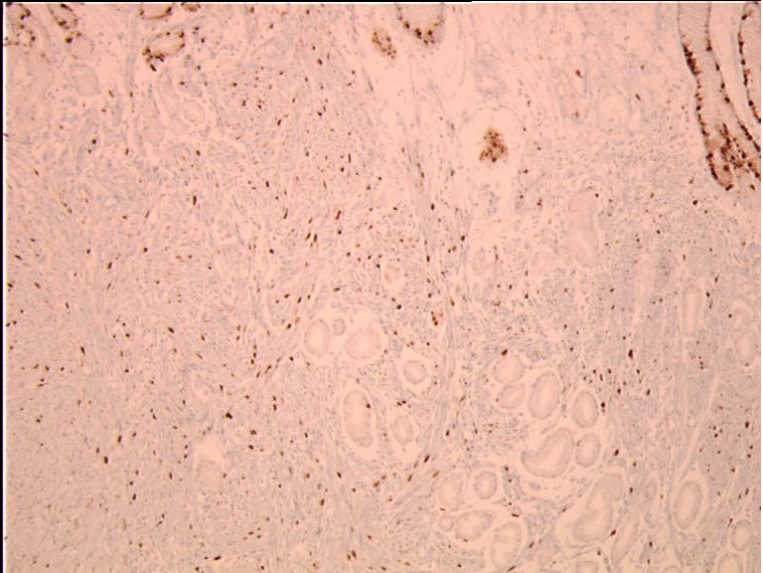
**The proliferative activity of the tumor infiltrate, identified by MiB-1, is approximately 20%**. There is a normal proliferative activity of epithelial cells in the bottom of foveolar gastric glands (MIB-1, original magnification × 100).

The polypous lesion, 1 cm in diameter, showed regular foveolar gastric glands and a diffuse spindle cell infiltrate in the mucosa. The spindle cells were arranged in parallel and whorl like bundles. The nuclei were elongated with plump ends and focally mild atypia (Fig. 1). There was an increased mitotic rate with 20 mitotic figures in 50 high power fields. In the immunoassay, the tumor cells stained strongly positive for SMA (Fig. 2) and negative for KIT, CD34, and S100. The proliferative activity, identified by MiB-1, was approximately 20% (Fig. 3). The spindle cell infiltrate was classified as an unusual intramucosal leiomyosarcoma of low grade malignancy. The diagnosis was confirmed by a referee pathologist. The tumor achieved the resection mark of the biopsy focally. In endosonographic monitoring 4 weeks later, there was no tumor residuum observed. Because of tumor malignancy, mucosectomy followed. The biopsy revealed a scar next to regular mucosa and lamina muscularis mucosae. Tumor residuum was not seen. In the literature were neither data of tumor treatment nor data with long time follow-up of intramucosal leiomyosarcoma available. The clinical outcome was not predictable. According to an expert of the European Sarcoma Study Group, a limited resection of the gastric antrum was recommended and done 4 weeks later. Reexamination revealed a chronic gastritis and a scar after mucosectomy, but no tumor residuum. Perigastric lymph nodes and a paracaval lymph node were free of tumor. A R0-resection with high safety of the resection margin was achieved. The endoscopic and endosonographic follow-up was inconspicuous since 3 years.

## Discussion

Leiomyosarcomas of the stomach are rare. They usually present in older age and are typically of high grade malignancy (WHO 2000). They arise from the smooth muscle of gastric wall and were mostly located in the submucosa. The histological diagnosis of a leiomyosarcoma is unequivocal on the basis of the immunohistochemical expression of SMA. At the time of primary diagnosis the tumor size is normally large. Complete tumor resection is the standard treatment. The reported case was exceptionally, clinical and histological. The leiomyosarcoma resembled a polyp of the gastric mucosa without criteria of a stromal tumor or signs of malignancy. The tumor was limited to the mucosa, showed mild nuclear atypia but a high proliferative activity. It is the unique intramucosal leiomyosarcoma of the stomach and the first gastric leiomyosarcoma described in a survivor of a retinoblastoma in childhood. Because of the young age of the patient, the high proliferative activity of the tumor and the visceral tumor site, a resection with large tumor free margins was striving. There is an increased risk for the development of a metachronous malignancy following hereditary retinoblastoma due to the prior treatment and/or genetic susceptibility of *RB1 *[1,2]. Alterations in *RB1 *are thoroughly investigated in soft tissue tumors [3]. Most often are osteosarcomas, followed by soft tissue sarcomas. One study reported about three patients with leiomyosarcoma of the soft tissue, in the radiation field of a primary malignancy in childhood, 11 to 13 years earlier [1]. A leiomyosarcoma of the liver was detected in a 39-year-old woman, who has been treated 37 years before, for hereditary retinoblastoma of the eye [4]. A leiomyosarcoma in the maxillofacial region, followed by a chorioncarcinoma 5 years later, was described in a long-term follow up after treatment of a bilateral retinoblastoma [5]. Visceral leiomyosarcoma of the urinary bladder is reported in two cases, 38 years and 47 years after hereditary retinoblastoma [6,7]. The patients had a tumor free survival of about 3 decades between retinoblastoma and second malignancies. This is in common with our case. Similar results were reported in a large cohort of retinoblastoma patients recently [2]. However, 15 out of 23 leiomyosarcoma occurred outside the radiation field of retinoblastoma. Most frequently were uterine leiomyosarcoma. It seems unlikely that the radiation exposure caused the leiomyosarcoma. Moreover a radiation – induced chromosome instability of single normal *RB1 *copy seems to be involved in tumor development. Radiation combined with chemotherapy was associated with a heightened risk for leiomyosarcoma in this study.

Since neither radiation nor chemotherapy treatment is reported in our case, primary genetic alterations, i.e. in *RB1 *may have a protooncogenetic effect on the development of secondary malignancies. As described earlier there is an increased risk to develop a third tumor.

## Conclusion

This is the unique report of an intramucosal gastric leiomyosarcoma and the first account of a gastric leiomyosarcoma after retinoblastoma in childhood. A careful clinical follow-up is advised because of increased risk of developing further metachronous malignancies.

## Consent

Written informed consent was obtained from the patient for publication of this case report and accompanying images. A copy of the written consent is available for review by the Editor-in-Chief of this journal.

## Competing interests

The authors declare that they have no competing interests.

## Authors' contributions

UP drafted the manuscript with review of the literature and took the microscopic imaging.

HG participated in the care of the patient, contributed the clinical data and revised the manuscript for intellectual content and given final approval of the version to be published.

Both of the authors read and approved the final manuscript.
